# Enhancing methane production from lignocellulosic biomass by combined steam-explosion pretreatment and bioaugmentation with cellulolytic bacterium *Caldicellulosiruptor bescii*

**DOI:** 10.1186/s13068-018-1025-z

**Published:** 2018-01-29

**Authors:** Daniel Girma Mulat, Silvia Greses Huerta, Dayanand Kalyani, Svein Jarle Horn

**Affiliations:** 1 Faculty of Chemistry, Biotechnology and Food Science, Norwegian University of Life Sciences, P.O.Box 5003, 1432 Ås, Norway; 2 Department of Chemical Engineering, University of Valencia, P.O.Box 46100, Valencia, Spain

**Keywords:** Anaerobic digestion, Biogas, Bioaugmentation, *Caldicellulosiruptor bescii*, Cellulolytic bacteria, Steam-explosion pretreatment, Birch, Lignocellulose

## Abstract

**Background:**

Biogas production from lignocellulosic biomass is generally considered to be challenging due to the recalcitrant nature of this biomass. In this study, the recalcitrance of birch was reduced by applying steam-explosion (SE) pretreatment (210 °C and 10 min). Moreover, bioaugmentation with the cellulolytic bacterium *Caldicellulosiruptor bescii* was applied to possibly enhance the methane production from steam-exploded birch in an anaerobic digestion (AD) process under thermophilic conditions (62 °C).

**Results:**

Overall, the combined SE and bioaugmentation enhanced the methane yield up to 140% compared to untreated birch, while SE alone contributed to the major share of methane enhancement by 118%. The best methane improvement of 140% on day 50 was observed in bottles fed with pretreated birch and bioaugmentation with lower dosages of *C. bescii* (2 and 5% of inoculum volume). The maximum methane production rate also increased from 4-mL CH_4_/g VS (volatile solids)/day for untreated birch to 9–14-mL CH_4_/g VS/day for steam-exploded birch with applied bioaugmentation. Bioaugmentation was particularly effective for increasing the initial methane production rate of the pretreated birch yielding 21–44% more methane than the pretreated birch without applied bioaugmentation. The extent of solubilization of the organic matter was increased by more than twofold when combined SE pretreatment and bioaugmentation was used in comparison with the methane production from untreated birch. The beneficial effects of SE and bioaugmentation on methane yield indicated that biomass recalcitrance and hydrolysis step are the limiting factors for efficient AD of lignocellulosic biomass. Microbial community analysis by 16S rRNA amplicon sequencing showed that the microbial community composition was altered by the pretreatment and bioaugmentation processes. Notably, the enhanced methane production by pretreatment and bioaugmentation was well correlated with the increase in abundance of key bacterial and archaeal communities, particularly the hydrolytic bacterium *Caldicoprobacter*, several members of syntrophic acetate oxidizing bacteria and the hydrogenotrophic *Methanothermobacter*.

**Conclusion:**

Our findings demonstrate the potential of combined SE and bioaugmentation for enhancing methane production from lignocellulosic biomass.

## Background

Anaerobic digestion (AD) of lignocellulosic biomass such as agricultural and wood residues for biogas production is attracting wide attention because of their abundance and environmental sustainability. Woody biomass in particular has comparative advantage over agricultural residues in terms of reduced transportation cost due to its high bulk density, possibility of year round harvest, and availability of well-established logistics [1]. In addition, biogas production from woody biomass may add value to the forest sector, which in recent years has experienced a rapid decline in the traditional pulp and paper industry [2]. Despite its potential for biogas production, woody biomass has a complex compositional and structural features making it generally resistance to biological degradation, a phenomenon known as biomass recalcitrance. Like other lignocellulosic biomass, woody biomass consists of three major structural biopolymers, namely, cellulose, hemicelluloses, and lignin. The cellulose microfibrils are locked in a matrix of intertwined hemicelluloses and lignin called lignin–carbohydrate complexes (LCC), forming a barrier for efficient biological deconstruction [3]. Thus, different strategies need to be employed to reduce biomass recalcitrance and thus increase the accessibility of lignocellulosic biomass to anaerobic microbial deconstruction.

Pretreatment is usually employed prior to AD for reducing biomass recalcitrance. Various pretreatment methods have been applied for enhancing the digestibility of lignocellulosic biomass, including physical, chemical, biological, or combinations of these techniques [4–6]. Depending on the type of pretreatment, several characteristics of biomass are altered including biomass composition, LCC, crystallinity, cellulose degree of polymerization, and accessibility (surface area, pore size, and pore volume) [3]. Steam explosion (SE) is considered as one of the most efficient pretreatment technologies and among the few pretreatments employed at industrial scale [7]. It reduces biomass recalcitrance due to the opening of the lignocellulosic fiber structure, reduction in fiber length, solubilization of hemicelluloses, and redistribution of lignin [8].

Biogas production from lignocellulosic biomass can also be enhanced by operating biogas digesters at high temperature. This is particularly important to improve the hydrolysis step, which is generally regarded as the slowest and, therefore, rate-limiting step during AD of particulate organic materials such as lignocellulosic substrates [9]. Thermophilic AD (55–70 °C) has a rate-advantage over mesophilic digestion (37 °C) due to higher hydrolysis coefficient and faster reaction rates [10, 11]. Additional benefits include increased degradation efficiency, increased biogas production, and improved reduction of pathogens [10, 12, 13].

Bioaugmentation can also be used to introduce specific microorganisms directly into biogas digesters to improve certain stages of the AD process [14]. Several biogas studies have shown that bioaugmentation with cellulolytic bacteria or bacterial consortia can increase the hydrolysis rate and consequently, enhanced the methane production from lignocellulosic substrates such as wheat straw [14–16], blends of DGS (distillers grains with solubles) and pig manure [17], blends of sludge, dried plant biomass from Jerusalem artichoke and pig manure [18], cellulosic waste material generated from sweet corn processing [19], cellulose and corn stover [20], and cattle manure [13]. Furthermore, the addition of hydrolytic/fermentative bacteria resulted in the production of higher concentrations of hydrogen which could promote the development of hydrogenotrophic methanogenesis, resulting in higher methane yields [17, 18, 21, 22].

Despite the potential of bioaugmentation for improving hydrolysis and ultimately, enhancing biogas production, their potential has not been fully realized, since the degradable carbohydrate fractions are often shielded by lignin in native substrates, and thus decreasing their accessibility to enzymatic and microbial deconstruction. For instance, most of the bioaugmentation studies described above used untreated lignocellulosic substrates and obtained only a maximum of 40% improvement in methane yield as a result of bioaugmentation. However, combined pretreatment and bioaugmentation to enhance methane production has achieved 210–246% increase in methane yields (Hu et al. [42]). Therefore, pretreatment followed by bioaugmentation seems like a very promising strategy to produce methane from lignocellulosic biomass. Moreover, bioaugmentation with the cellulolytic bacterium *Caldicellulosiruptor bescii* has not been reported before despite the ability of *C. bescii* to use a wide range of substrates, including cellulose, hemicellulose, and lignocellulosic substrates and ferment C6 and C5 sugars simultaneously [23, 24]. *C. bescii*, unlike most other cellulolytic bacteria, utilizes distinctive cellulolytic enzymatic systems in which the individual cellulases secreted are multimodular, containing multiple binding and catalytic domains [25]. These distinctive enzymatic mechanisms could synergize with other cellulases secreted by indigenous anaerobic bacteria. Therefore, all these properties make *C. bescii* a promising candidate for bioaugmentation.

This study employed SE pretreatment and bioaugmentation with cellulolytic bacterium *C. bescii* under thermophilic conditions (62 °C) to reduce biomass recalcitrance, improve hydrolysis, and increase biogas yields of a lignocellulosic substrate. Birch was used as a model lignocellulosic substrate, which is a representative of hardwood species widely distributed and available in the temperate regions of the northern hemisphere. In addition, possible changes in bacterial and archaeal community structures in the samples collected at the end of the experiment were studied by amplicon sequencing of the 16S rRNA gene.

## Methods

### Chemicals

Glucose, mannose, galactose, xylose, and arabinose were obtained from Fluka (Milwaukee, WI) and Alfa Aesar (Ward Hill, MA). The reducing reagents (cysteine and glutathione) were purchased from Sigma-Aldrich (St. Louis, MO). Unless otherwise specified all the other reagents used in this study were of analytical grade and obtained from Sigma-Aldrich (St. Louis, MO).

### Inoculum and anaerobic medium

The original microbial inoculum used in this experiment was collected from a full-scale continuously stirred tank reactor (CSTR) (Nordre Follo Wastewater Treatment Plant, Vinterbro, Norway) running with sewage sludge and food waste at thermophilic temperature (~ 62 °C). This inoculum was used to run sequential batch bottles fed with untreated and steam-exploded birch. The digestate from the sequential batch bottles was used as inoculum, which has a dry matter (DM) content of 3.8%, the volatile solid (VS) content of 2.1%, and the pH of 7.8. Anaerobic medium was prepared from a mixture of mineral buffer solution, potassium hydrogen phosphate, trace elements, selenite, and vitamins according to Angelidaki et al. with a slight modification [26]. Briefly, a stock solution of mineral buffer solution was prepared (concentration/L): 100-g NH_4_Cl, 10-g NaCl, 10-g MgCl_2_·6H_2_O, and 5-g CaCl_2_·2H_2_O. A stock solution (1 L) of potassium hydrogen phosphate was prepared from 200-g K_2_HPO_4_·3H_2_O. A stock solution of the vitamin solution was prepared (concentration/L): 2-mg biotin, 2-mg folic acid, 10-mg pyridoxine–HCl, 5-mg thiamine–HCl, 5-mg riboflavin, 0.1-mg vitamin B12, 5-mg nicotinic acid, 5-mg *p*-aminobenzoic acid, 5-mg lipoic acid, and 5-mg calcium pantothenate. The trace element and selenite solution contained (L^−1^): 2-g FeCl_2_·4H_2_O, 0.05-g ZnCl_2_, 0.05 MnCl_2_·4H_2_O, 0.05-g H_3_BO_3_, 0.05-g CoCl_2_·6H_2_O, 0.038-g CuCl_2_·2H_2_O, 0.05-g AlCl_3_, 0.092-g NiCl_2_·6H_2_O, 0.05-g Na_2_MoO_4_·2H_2_O, 0.5-g ethylenediaminetetraacetate, 1-mL concentrated HCl, and 0.1-g Na_2_SeO_3_·5H_2_O. A mixture of anaerobic medium was prepared in 975 mL of distilled water from stock solutions of mineral buffer solution (10 mL), potassium hydrogen phosphate solution (2 mL), vitamin solution (1 mL), and trace element and selenite solution (1 mL). The mixture of all ingredients was boiled (except cysteine, bicarbonate, and sulfide), and then, it was cooled to room temperature under 80% N_2_:20% CO_2_ gas mixture to maintain neutral pH. The solution was dispensed under the same gas atmosphere into serum vials and autoclaved. The cysteine and sulfide were sterilized separately by filtration. The medium was reduced using (L^−1^) 0.5-g cysteine and 0.5-g Na_2_S, and then, Na_2_CO_3_ (2.6 g/L) was added. The final pH of the anaerobic medium was 7.0.

### Raw material

Birch (*Betula pubescens*) wood chips originated from a tree harvested in 2009 in Norway (60.7°North, 10.4°East). The birch tree trunk was debarked and chipped to produce 20–30 mm chip fractions. These fractions were dried at room temperature and subsequently milled to pass a sieve of 6 mm (SM 2000, Retsch, Haan, Germany) and stored at room temperature and dry conditions. The DM and VS contents of the dried birch were 94.9 and 94.8% (fresh biomass weight), respectively. DM is the sum of VS and ash.

### Cellulolytic bacteria culture used for bioaugmentation

The strain *Caldicellulosiruptor bescii* DSM 6725 was revived from the freeze-dried culture that was obtained from the DSMZ (Braunschweig, Germany). It was grown in DSMZ 516 medium with the following modification. The mineral solution contained (L^−1^): 0.5-g NH_4_Cl, 0.5-g KH_2_PO_4_, 0.33-g KCl, 0.33-g MgCl_2_·6H_2_O, 0.14-g CaCl_2_·2H_2_O, 0.5-g yeast extract, 5-g cellobiose, 0.5-mL resazurin (0.05% w/v), 5-mL vitamin solution, and 1-mL trace-element solution. The vitamin solution contained (L^−1^): 4-mg biotin, 4-mg folic acid, 20-mg pyridoxine–HCl, 10-mg thiamine–HCl, 10-mg riboflavin, 10-mg nicotinic acid, 10-mg calcium pantothenate, 0.2-mg vitamin B12, 10-mg *p*-aminobenzoic acid, and 10-mg lipoic acid. The trace-element solution contained (L^−1^): 1.5-g FeCl_2_·4H_2_O, 0.07-g ZnCl_2_, 0.1 MnCl_2_·4H_2_O, 0.006-g H_3_BO_3_, 0.19-g CoCl_2_·6H_2_O, 0.002-g CuCl_2_·2H_2_O, 0.024-g NiCl_2_·6H_2_O, and 0.036-g Na_2_MoO_4_·2H_2_O. The mixture of all ingredients was prepared (except cysteine, carbonate, cellobiose, and sulfide) and boiled, and then, it was cooled to room temperature under CO_2_ gas. The solution was dispensed under the same gas atmosphere into serum vials and autoclaved. The cellobiose, cysteine, and sulfide were sterilized separately by filtration using a 0.22-μm-pore-size sterile filter (Millipore Filter Corp., Bedford, MA). The medium was reduced using (L^−1^) 0.5-g cysteine and 0.5-g N_2_S, and then, Na_2_CO_3_ (1 g/L) was added. The final pH was 7.0. The cultures were incubated at 65 °C for 3 days under static conditions. Cell growth was monitored by optical density (680 nm) using a spectrophotometer (Hitachi U-1900, Hitachi High-Technologies Corporation, Tokyo, Japan). After 3 days of incubation (OD680 of approximately 0.54), the *C. bescii* culture was harvested in the exponential phase and used as a supplementary inoculum (bioaugmentation) to set up the biogas batch experiments described below.

### Steam-explosion (SE) pretreatment

SE pretreatment was conducted using a steam-explosion unit designed by Cambi AS (Asker, Norway) situated at Norwegian University of Life Science. In a previous study [6], the optimal steam-explosion conditions of birch for biogas production were found to be pretreatment at 210 °C and 10-min residence time. Therefore, we used the same pretreatment conditions in this study. The pretreated material was stored in plastic bags at 4 °C until the start of the biogas experiment. The DM and VS contents of the steam-exploded birch were 35.0 and 34.9%, respectively.

### Batch experiments to test the biogas production potential of steam-exploded birch with and without applied bioaugmentation

The potential of steam exposition (SE) and bioaugmentation for enhancing biogas production of birch was investigated in 120-mL batch bottles with working volume of 70 mL. A total of 11 sets of batch bottles were prepared (Table 1). The bottles running with steam-exploded birch with applied bioaugmentation (pretreated + 2–15% v/v) received the same amount of steam-exploded material and inoculum, and different volumes of *C. bescii* culture, corresponding to 2, 5, 10, and 15% v/v of the volume of the inoculum. Non-bioaugmented control bottles fed with steam-exploded birch (pretreated + 0%) were prepared with the same amount of steam-exploded material and inoculum. Non-bioaugmented bottles fed with untreated birch (untreated + 0%) were also prepared to compare the process performance with bottles fed with steam-exploded birch. All the substrate-amended bottles received equal amount of inoculum, substrate (based on VS), and anaerobic medium. The inoculums-to-substrate ratio was 2:1 (based on VS basis) as suggested by Holliger et al. [27]. In addition, negative controls with only inoculum (inoculum + 0%) as well as inoculum and *C. bescii* culture (inoculum + 2–15%), corresponding to 2, 5, 10, and 15% v/v of the volume of the inoculum, were prepared to correct for the endogenous biogas production. These negative controls received water instead of substrate to maintain the same working volume as the substrate-amended reactors. The bottles were flushed with nitrogen gas for a few minutes and sealed with septum and aluminum caps to maintain anaerobic conditions. All the experiments were conducted in triplicate inside a shaker (Multitron Standard, Infors HT, Switzerland) under thermophilic conditions (62 °C, 120 rpm). The biogas experiment run for 50 days and terminated when the daily biogas rate on three consecutive days was below 1% [27].

**Table 1:** Biological methane potential (BMP) test experimental setup

Name	Substrate	Inoculum	*C. bescii* culture (v/v), %^c^
Untreated + 0%	Untreated birch	+^a^	–^b^
Pretreated + 0%	Pretreated birch	+	–
Pretreated + 2%	Pretreated birch	+	2
Pretreated + 5%	Pretreated birch	+	5
Pretreated + 10%	Pretreated birch	+	10
Pretreated + 15%	Pretreated birch	+	15
Inoculum + 0%	–	+	–
Inoculum + 2%	–	+	2
Inoculum + 5%	–	+	5
Inoculum + 10%	–	+	10
Inoculum + 15%	–	+	15

^a^The positive sign represents that all the bottles received the same amount of inoculum

^b^The negative sign represents that these bottles did not receive the indicated materials (*C. bescii* culture and/or substrate)

^c^The volume of *C. bescii* culture divided by the total volume of the solution (70-mL working volume). Optical density (680 nm) of the culture was 0.54

### Acid-insoluble lignin and carbohydrate analysis

Samples for carbohydrate and acid-insoluble lignin content analysis were prepared using a standard NREL two-stage acid hydrolysis protocol [28]. Acid hydrolysis generates soluble sugars and acid-insoluble lignin residues, where the later was dried overnight at 105 °C in an oven (Heratherm oven, Thermo Scientific, MA, USA) and weighed to obtain the acid-insoluble lignin (Klason lignin) content. The soluble sugars were analyzed for carbohydrate constituents by high-performance anion-exchange chromatography with pulsed amperometric detection (HPAEC-PAD) (Dionex ICS-3000, Dionex, Sunnyvale, CA, USA). Separation of soluble sugars was achieved utilizing a CarboPac-PA1 2 × 250 mm analytical column equipped with a CarboPac PA1 2 × 50 mm guard column (both from Dionex, Sunnyvale, CA), operated at 30 °C, with Milli-Q water as a mobile phase with a flow rate of 0.250 mL/min. The total run time was 35 min. External calibration curves were established using the standard solutions of arabinose, galactose, glucose, mannose, and xylose. The standard solutions were prepared from their corresponding monosaccharide (> 99%) obtained from Sigma-Aldrich.

### Biogas composition and calculation

The biogas production was periodically monitored by measuring the gas pressure in the headspace of the batch bottles using a digital pressure transducer (GMH 3161, Greisinger Electronic, Regenstauf, Germany). After recording the pressure in the batch bottles, the overpressure was released by penetrating the septum with a needle. To avoid excessive dissolution of CO_2_ with possible effects on pH, the overpressure was always kept below 200 kPa (Holliger et al. [27]). The biogas composition (CH_4_ and CO_2_) was analyzed by gas chromatography (GC) using a gas chromatograph (3000A Micro GC, Agilent Technologies, Wilmington, USA) equipped with a thermal conductivity detector (TCD). For separation of gases, two parallel capillary columns containing different coatings (MolSieve 5 Å PLOT, 10 m × 0.32 mm × 12 μm and PLOT Q, 10 m × 0.32 mm × 10 μm) were used. The operational temperature of sample inlet was kept the same for both columns at 60 °C. The operational injector and column temperatures for the MolSieve 5 Å PLOT were 90 and 70 °C, respectively, while the other column was operated at 50 and 45 °C, respectively. Both columns were connected to TCD with helium applied as a carrier gas. Certified standard mixture of CO_2_ and CH_4_ in nitrogen (AGA, Norway) was used for calibration. Using the measured overpressure, headspace volume of the bottles and methane concentration as input, the ideal gas law was applied for calculating the volume of methane produced. The volume of the methane produced for substrate-amended bottles with and without supplied bioaugmentation was reported after correcting the background methane production from the negative controls (inoculum with applied bioaugmentation and inoculum alone, respectively). The volume of methane produced was reported at standard temperature and pressure (0 °C and 1 atm). The average results of the biological triplicates are presented with standard deviations.

### Other analytical methods

The DM and VS of the substrates were analyzed according to the standard methods (APHA, 2005).

### Microbial community analysis

At the completion of the study, the samples collected from all batch bottles were analyzed for their microbial community composition. The genomic DNA was extracted from the samples stored at − 20 °C. 1 mL of the sample was centrifuged at 10,000×*g* for 5 min to remove the supernatant. Afterwards, the pellet was resuspended in 300 µL of S.T.A.R. buffer (Roche Diagnostics, Penzberg, Germany) to stabilize the nucleic acids in the sample. Cells were mechanically disrupted in a MagNa Lyser instrument (Roche Diagnostics GmbH, Mannheim, Germany) by adding 0.25 g of acid-washed glass beads and bead-beating twice at 6500 rpm and room temperature for 20 s each time. Thereafter, the sample was centrifuged at 13,000×*g* for 5 min to recover the DNA from the supernatant. The DNA was extracted using the MagMidi kit (LGC Genomics, UK) for the KingFisher Flex robot (ThermoFisher Scientific, Wilmington, USA) according to the manufacturer’s protocol, and its concentration was measured by Qubit fluorometer with Quant-iT dsDNA Br assay kit (Invitrogen, USA). The DNA quality was evaluated with the Nanodrop ND 1000 spectrophotometer (ThermoFisher Scientific, Wilmington, USA).

The extracted DNA was amplified using polymerase chain reaction (PCR) primers Pro341F/Pro805R: 5′-CCTACGGGNBGCASCAG-3′/5′-GACTACNVGGGTATCTAATCC-3′ [29], which target the V3–V4 hypervariable regions of bacterial and archaeal 16S rRNA gene. The PCR mixture (25 µL) contained 2.5 µL of DNA template (5 ng/µL), 12.5 µL of iProof HF Master Mix (BIO-RAD, USA), 0.625 µL of each primer (10 µM), and 8.75 µL nuclease free water. The PCR cycles were: an initial denaturation step at 98 °C for 3 min, followed by 30 cycles consisting of 98 °C for 10 s, 55 °C for 30 s, and 72 °C for 30 s, with a final elongation step at 72 °C for 5 min. Agencour tAMPure XP (Beckman Coulter, USA) was used for purification of the PCR products. The size and purity of amplicons were checked by electrophoresis on 1% w/v agarose gel. Nextera XT DNA Library Preparation Kit (Illumina, San Diego, CA, USA) was used to index the PCR-amplified samples, according to manufacturer’s protocol. The barcoded amplicons were quantified using a Qubit fluorometer with Quant-iT dsDNA BR assay kit (Invitrogen, USA), and each amplicon was adjusted to equimolar concentration according to the Illumina protocol for 16S Metagenomic Sequencing Library Preparation. Finally, Illumina MiSeq sequencer (Illumina, San Diego, CA, USA) was used to sequence the denatured DNA using MiSeq reagent kit v3 (600-cycle).

Sequence data from the samples were analyzed with Quantitative Insight Into Microbial Ecology (QIIME) 1.9.1 software package [30]. Previously, the downstream analysis, paired-end reads from every sample were merged using PEAR program [31], followed by quality filtering using PRINSEQ [32] at mean quality score of 30 and a minimum length of 350 bp. The primers sequences were trimmed by Mothur [33]. Chimeric sequences were removed, followed by clustering into operational taxonomic sequences (OTUs) at 97% sequence identity by USEARCH [34, 35], which is implemented in QIIME, using the Greengenes database *gg_13_8* [36]. Raw sequences are made available at the Sequence Read Archive (SRA) with the accession numbers SRR5921530–SRR5921558 as part of BioProject PRJNA394663.

### Data analysis

Kinetic parameters such as *B*_0_, *R*_max,_ and *λ* were estimated by fitting the experimental data, obtained from the batch assays, to the modified Gompertz equation [37]:

1 $$ B(t) = B_{0} \exp \left\{ { - \exp \left[ {\frac{{R_{\text{max} } e}}{{B_{0} }}} \right]\left( {\lambda - t} \right) + 1} \right\} $$

where *B*(*t*) = is the cumulative CH_4_ yield at incubation time *t* (mL CH_4_/g VS), *B*_0_ = the CH_4_ potential (mL CH_4_/g VS), *R*_max_ = the maximum CH_4_ production rate (mL CH_4_/g VS/day), *λ* = the lag phase (d), and *e* = Euler’s constant. To evaluate the accuracy of predictions, the coefficient of determination (*R*^2^) and root mean square error (RMSE) were calculated. Microsoft PowerPoint 2010 (Microsoft, WA 01060, USA) was used for non-linear fitting and corresponding statistical analysis.

Microsoft PowerPoint 2010 (Microsoft, WA 01060, USA) and Origin 8.0 (Origin Lab, WA 01060, USA) were used for graphing.

## Results and discussion

### Feedstock characteristics

The DM content decreased from 94.8% in untreated birch to 35.0% in steam-exploded birch, as steam is added to the biomass during the pretreatment. Since the pH of steam-exploded birch was very low (3.0), it was adjusted to 7.5 by adding NaOH prior to the biogas experiments. The low pH can be explained by the release of organic acids from the degradation of hemicelluloses during SE pretreatment [38]. The VS content of the untreated and pretreated birch was similar (99.8% of DM).

The content of cellulose, hemicelluloses, and Klason lignin in the untreated and steam-exploded birch samples is summarized in Table 2. The proportion of cellulose and Klason lignin in the steam-exploded birch increased, while the amount of hemicelluloses (mainly xylan) was reduced. Such large reductions in the content of hemicelluloses have been reported in a previous study of birch pretreated with SE [6]. The employed high temperature and acidic condition (released organic acids) can catalyze the hydrolysis of hemicelluloses and further degradation into lower molecular weight (LMW) compounds. These LMW compounds (such as furfural) can repolymerize to form lignin-like material termed “pseudo-lignin” [39]. Thus, the higher Klason lignin content of the steam-exploded sample compared to the starting material (untreated birch) is partly due to the formation of pseudo-lignin, which is known to remain as acid-insoluble residues during the standard NREL two-stage acid hydrolysis protocol. It should be noted that loss of volatile LMW compounds formed from hemicellulose during steam-explosion pretreatment also would contribute to the higher content of the Klason lignin (and cellulose) in the steam-exploded material.

**Table 2:** Chemical composition of untreated and steam-exploded birch

Birch	Composition (%)					
	Arabinan	Galactan	Glucan	Xylan	Mannan	Klason lignin
Untreated	1.1 ± 0.03	1.60 ± 0.05	37.6 ± 2.13	16.2 ± 0.72	2.8 ± 0.02	30.1 ± 1.10
Pretreated^a^	0.2 ± 0.01	0.9 ± 0.03	44.3 ± 2.09	9.7 ± 0.10	1.7 ± 0.04	39.4 ± 1.40

The amounts of all components are expressed as a percentage of dry matter. The amount of carbohydrates was calculated using the mass of anhydrous sugar

^a^The steam-exploded material was not washed and represents the whole slurry obtained after the pretreatment

### Enhanced biogas production by combined SE and bioaugmentation

**Fig. 1:**
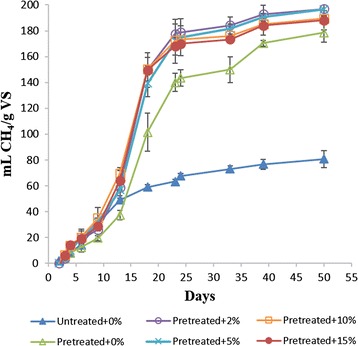
Cumulative methane production reactors fed with untreated birch without bioaugmentation (closed triangles), steam-exploded birch without bioaugmentation (open triangles, as well as steam-exploded birch with applied bioaugmentation at different *C. bescii* loadings (2% v/v open circles; 5% v/v crosses; 10% v/v open squares; and 15% v/v closed circles)

The effect of SE pretreatment on methane production was investigated using batch experiments run with untreated and steam-exploded birch for 50 days (Fig. 1). The data presented in Fig. 1 are the net methane production from the birch substrate after correcting for the background methane production from the inoculum or inoculum with *C. bescii*. SE pretreatment clearly influenced the methane production rate and yield, and the lag phase (Table 3). Following SE pretreatment, the final methane yield was increased from 81- to 179-mL CH_4_/g VS (increased by 118%). While the initial rate of methane production seems similar for both pretreated and untreated materials, the rate substantially increased in bottles fed with pretreated material after day 13. The maximum rate of methane production also increased from 4.2-mL CH_4_/g VS/day for bottles fed with untreated birch to 9.9-mL CH_4_/g VS/day for steam-exploded birch (Table 3). Although the lag phase was relatively longer for bottles fed with pretreated birch, the rate and yield of methane production had significantly increased following SE. Thus, SE had improved the accessibility of the birch material to anaerobic bacteria. SE has been shown to result in hemicelluloses removal, lignin relocalization with some structural modification together with broken fiber and porous surface materials [8]. Such transformations of biomass could contribute to reduction of biomass recalcitrance and explain the higher methane yield obtained from the stream-exploded birch in this study.

**Table 3:** Parameters of modified Gompertz model fitting experimental data

Bottles	Measured CH_4_ yield, mL CH_4_/g VS	Calculated values from the model				
		CH_4_ yield, mL CH_4_/g VS	max CH_4_ rate, mL CH_4_/g VS/day	Lag phase, day	*R* ^2a^	RMSE^a^
Untreated + 0%	81 ± 7	79 ± 6	4.2 ± 1	2.20 ± 0.66	0.9892	3.52
Pretreated + 0%	179 ± 7	177 ± 6	9.9 ± 2	8.00 ± 0.32	0.9899	8.11
Pretreated + 2%	197 ± 9	197 ± 8	15.5 ± 3	8.19 ± 1.14	0.9896	9.52
Pretreated + 5%	196 ± 5	196 ± 5	13.3 ± 4	7.25 ± 2.05	0.9902	8.89
Pretreated + 10%	189 ± 2	189 ± 2	13.2 ± 2	6.47 ± 1.22	0.9896	8.87
Pretreated + 15%	188 ± 8	188 ± 7	13.9 ± 2	7.26 ± 0.72	0.9878	9.66

^a^*R*^2^ and RMSE were calculated from the average values of the measured methane production during 50 days and the one calculated using the model

The effect of combined SE and bioaugmentation with *C. bescii* on methane production was also investigated (Fig. 1). The methane yield had increased significantly (by 130–140%) by combined SE pretreatment and bioaugmentation in comparison with bottles fed with untreated substrate (Table 3). The maximum methane production rate was increased from 4.2-mL CH_4_/g VS/day for bottles fed with untreated birch to 13.2–15.5-mL CH_4_/g VS/day for all bottles running with steam-exploded material and bioaugmented with *C. bescii*.

The bottles with and without bioaugmentation (0–15% v/v loading of *C. bescii*) were also compared to evaluate the effect of bioaugmentation alone on methane production (Fig. 1). The methane yield was similar during the first 3 days with and without bioaugmentation but higher in the former afterwards. The enhancement in methane yield by bioaugmentation alone reached between 38 and 48% at the timepoint, where more than 60% of methane was produced (day 18). This enhancement was reduced later and were 5–10% on day 50. The highest methane improvement on day 50 was 10% in bottles bioaugmented with lower dosages of *C. bescii* (2 and 5%). The maximum methane production rates were slightly improved and the lag phase periods were slightly shorter in bioaugmented bottles compared to the non-bioaugmented bottles. The observed positive effects of bioaugmentation may indicate an enhanced hydrolysis of steam-exploded materials by *C. bescii* and subsequent improvement in acidogenesis, acetogenesis, and finally methanogenesis. *C. bescii* was isolated from thermal springs of Kamchatka in Russia [40] and has been proven to be capable of hydrolyzing a variety of polysaccharides, including crystalline cellulose and untreated plant biomass [23, 24].

Solubilization of the carbohydrate fraction of the untreated and steam-exploded birch was compared among the batch bottles using Eq. (2). This equation estimates the fraction of particulate chemical oxygen demand (PCOD) that is converted to soluble material (SCOD). The COD equivalent of the produced methane was used to represent the SCOD. As suggested by Angelidaki et al. [26], the compositional analysis data such as the cellulose, hemicelluloses and lignin content can be used for calculating the COD of particulate substrates like birch instead of COD measurement by standard chemical methods [26]. This is because of the difficulty to obtain reliable COD measurements from heterogeneous particulate materials:

2 $$ {\text{Extent of solublization}}\, \left( \% \right) = \frac{\text{SCOD}}{\text{PCOD}} $$

where SCOD is the soluble COD, equivalent of the CH_4_ produced; PCOD is the particulate COD in the untreated and pretreated birch.

**Fig. 2:**
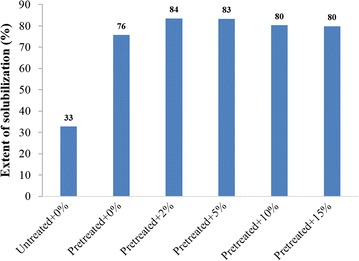
Extent of solubilization in the batch bottles fed with untreated birch without bioaugmentation, steam-exploded birch without bioaugmentation and steam-exploded birch with applied bioaugmentation at different *C. bescii* loadings from 2 to 15% v/v

The extent of solubilization of the untreated birch and pretreated birch with and without the applied bioaugmentation is presented in Fig. 2. It clearly shows that the solubilization was increased by more than twofold when birch was pretreated. The solubilization was also increased when combined SE pretreatment and bioaugmentation was employed in comparison with pretreatment alone. The increase in solubilization is consistent with the observed improvement in methane production (Fig. 1 and Table 2).

Biogas intensification by bioaugmentation with cellulolytic bacteria for improving hydrolysis has been reported in several biogas studies from untreated lignocellulosic substrates [15, 16] and only few studies from pretreated materials [41, 42]. In a batch experiment fed with untreated straw, bioaugmentation increased the methane yield by 27% in a 4% amended enrichment culture obtained from sheep rumen fluid containing cellulolytic bacteria [15]. In another bioaugmentation experiment with *Clostridium cellulolyticum*, long adaptation period (more than 10 days) and high amount of culture (25%) were required to enhance the methane yield by 7.6–13% [16]. In another recent study [43], individual and mixed cultures of *Ruminococcus flavefaciens 007C*, *Pseudobutyrivibrio xylanivorans Mz5T*, *Fibrobacter succinogenes S85,* and *Clostridium cellulovorans* were used to enhance methane production of brewer spent grain. The highest methane improvement reached up to 18% when *P. xylanivorans Mz5T* was used for bioaugmentation, whereas the other hydrolytic bacteria increased the methane yield by only 5–7%. Despite the positive effects of bioaugmentation on methane production from untreated lignocellulosic substrates, the methane yield in bioaugmented bottles is rather low in comparison with the theoretical methane expected from the carbohydrate composition of the substrates. Thus, combination of pretreatments and bioaugmentation strategies seems more effective for the conversion of the carbohydrates in the biomass into methane, as demonstrated in this study (up to 140% improvement in methane yield). It should be noted that SE pretreatment contributed to the major share of methane enhancement by 118%, while bioaugmentation notably increased the initial methane production rate by up to 44%.

Other few biogas studies also demonstrated the beneficial effects of combined pretreatment and bioaugmentation for biogas production [41, 42]. Combined thermal pretreatment (150 °C for 2 h) and bioaugmentation with an enriched culture containing lignocellulolytic microorganisms (*Clostridium stercorarium* and *Bacteroides cellulosolvens*) can substantially increase the methane yield of sludge by up to 246% compared to the control (untreated sludge), whereby the thermal pretreatment contributed the major share of methane enhancement by 223% [42]. In another study by Sträuber et al. [41], combined calcium hydroxide pretreatment (10% w/w of Ca(OH)_2_ per fresh weight of straw; stored at for 24 h at 22 °C) and two alkali-tolerant, lignocellulolytic environmental enrichment cultures, was employed to improve biogas production from wheat straw. The methane potential of the pretreated straw with and without bioaugmentation was 36% higher than that of untreated straw. Bioaugmentation only accelerated the methane production rate during the first week without enhancing the final methane yield. Overall, this and previously published studies supported the beneficial effects of combined pretreatment and bioaugmentation with cellulolytic bacteria for reducing biomass recalcitrance and improving hydrolysis, which contributed to ultimately improved methane production.

### Microbial community analysis

**Fig. 3:**
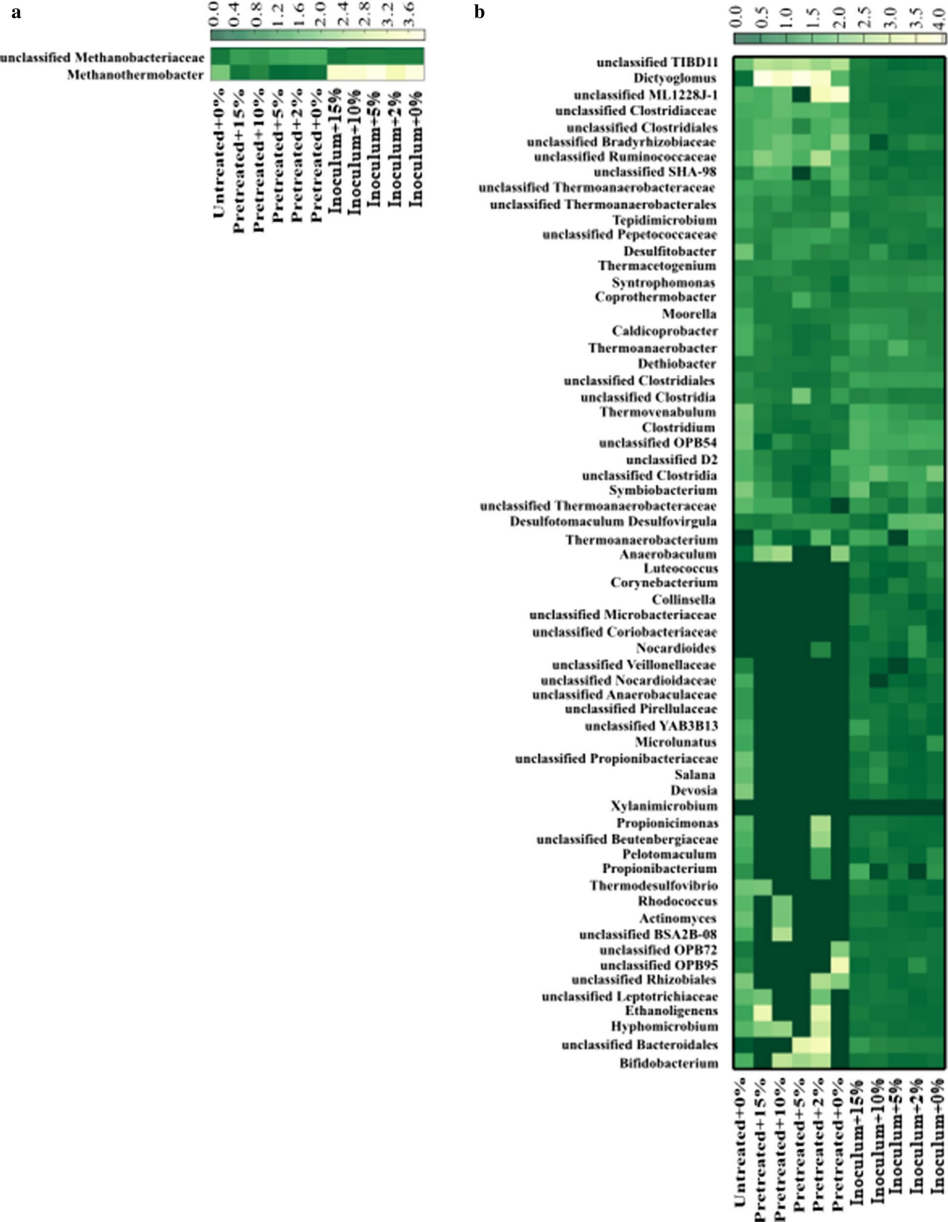
Heat maps of relative abundance (≥ 0.1%) of archaea (**a**) and bacteria community (**b**). Color scale refers to the OTU abundance and is reported on top of the two figures

**Fig. 4:**
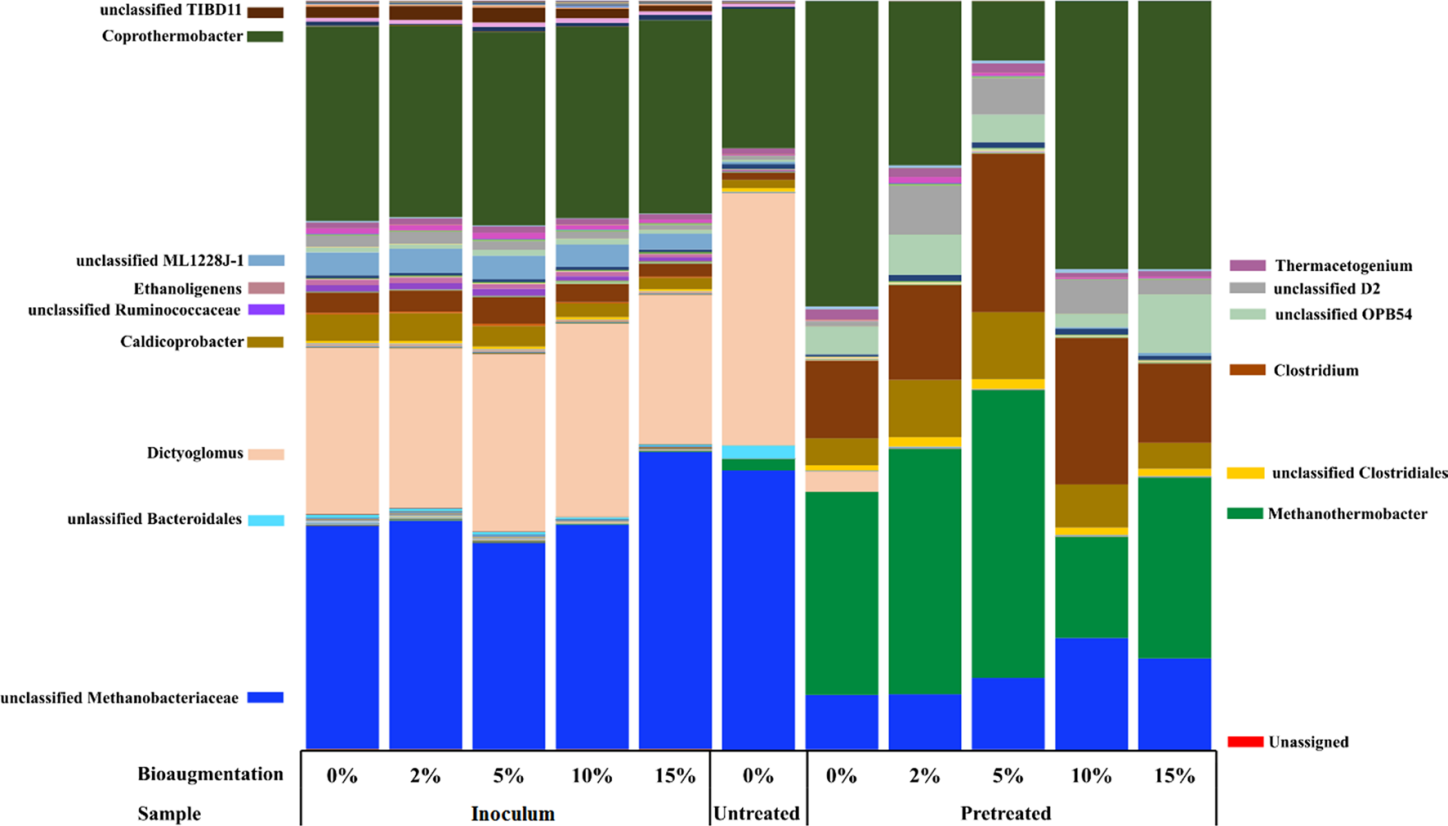
Relative abundance of bacterial and archaeal OTUs at genus level. Taxonomic groups with relative abundance lower than 1% were excluded from the plot legend flanking the bars

**Fig. 5:**
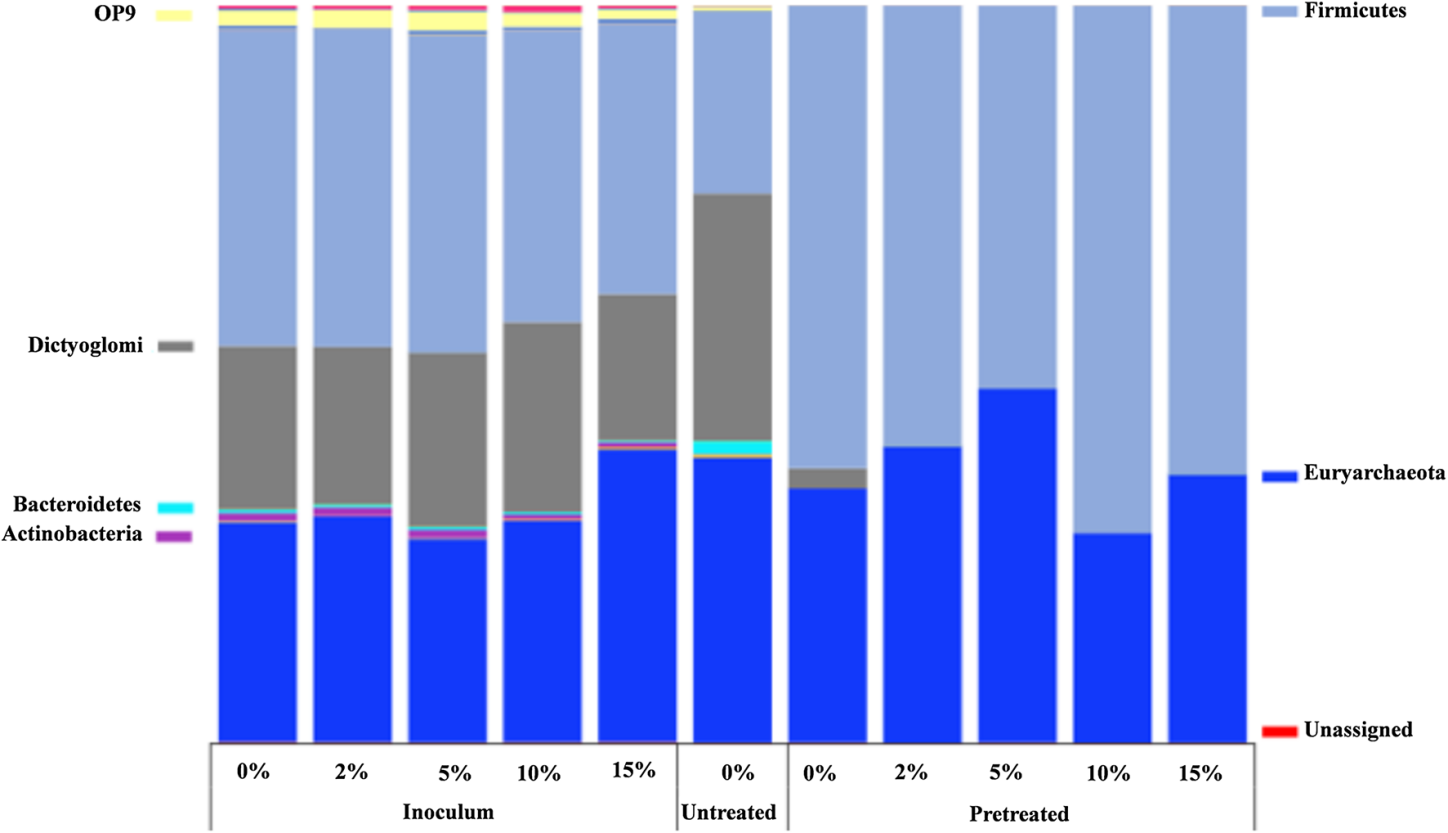
Relative abundance of bacterial and archaeal OTUs at phylum level. Taxonomic groups with relative abundance lower than 1% were excluded from the plot legend flanking the bars

The bacterial and archaeal communities in the samples from thermophilic bottles were analyzed by amplicon sequencing of 16S rRNA genes. As shown in Fig. 3, a total of 66 genera were identified which harbored ≥ 0.1% of the reads in one or more of the sequences. Of these, 15 genera were found to be highly abundant comprising at least 1% in at least one reactor (Fig. 4). The relative abundance of the OTUs comprising at least 1% in at least one reactor at the phylum level is shown in Fig. 5. The dominant bacterial phyla in all thermophilic bottles fed with pretreated substrate was *Firmicutes* (50–70% of total reads), whereas *Firmicutes* (23–40%) and *Dictyoglomi* (20–34%) dominated the bottles fed with untreated birch and without substrate (inoculum) (Fig. 5). Members of *Firmicutes* have been repeatedly identified as the main phyla in various anaerobic digesters along with *Bacteroidetes* [44–52]. Members of the phylum *Bacteroidetes* were detected in only the bottles fed with untreated birch at lower abundance (1.7%). The dominance of *Firmicutes* and absence of *Bacteroidetes* in all the thermophilic bottles except those fed with untreated birch suggest the importance of the former for the degradation of lignocellulosic biomass under thermophilic conditions. In a previous study of biogas digesters treating manure and straw, the relative abundance of *Bacteroidetes* decreased significantly from 13.2 to 16.6 to 0.4% with increase in operating temperature from 44 to 52 °C [53], whereas the relative abundance of *Firmicutes* increased during the increase in operating temperatures [53]. This suggests a competitive advantage of *Firmicutes* over *Bacteroidetes* under thermophilic conditions. The dominance of *Dictyoglomi* in all other samples except those from bottles running with pretreated material is less clear. Members of *Dictyoglomi* are known to have a cellulolytic activity [54] and have been detected previously in thermophilic biogas digesters fed with food waste (Hagen et al. [55]).

There are also other members of bacteria present in some of the biogas bottles with minor abundance (Fig. 5). For example, phylum “*Atribacteria*” *OP9* lineage was present in inoculum and those bottles fed with untreated birch at low abundance (about 2%) compared to *Dictyoglomi* and *Firmicutes*. Members of *OP9* have been observed in few thermophilic biogas digesters [55, 56] and suggested to hydrolyze complex carbohydrates such as cellulose and hemicellulose [57]. *Proteobacteria* was detected at about 0.1% in inoculum and bottles fed with untreated substrate, with all the members belonging to the known glucose utilizing *Alphaproteobacteria*.

Archaeal sequences detected in all the thermophilic bottles belonged to only the hydrogenotrophic *Methanobacteriaceae,* accounting for 30–48% of the total sequence reads (Fig. 5). Interestingly, sequences affiliated to acetoclastic methanogens were below the detectable level in any of the bottles, suggesting that acetoclastic methanogens are less important for methane production in anaerobic digesters above 60 °C [12]. A previous study showed that the hydrogenotrophic *Methanothermobacter* thrived when the operating temperature of biogas digesters was increased from 55 to 65 °C, whereas the mixotrophic *Methanosarcina* were no longer detectable at 65 °C [12]. In the absence of acetoclastic methanogens, methane production from acetate follows a two step pathway, i.e., acetate oxidation to CO_2_ by the syntrophic acetate oxidation (SAO) pathway followed by the reduction of CO_2_ into methane by hydrogenotrophic methanogenesis (HM). In the previous biogas digesters operated at temperatures above 60 °C [12], SAO-HM played key role for the conversion of acetate into methane. This study also supported the dominance of SAO-HM for methane production at high temperature (see the discussion below).

The abundance of the bacterial and archaeal community hardly changed in all the negative controls without substrate (inoculum with or without *C. bescii*) (Figs. 3, 4). This is not surprising as the remaining substrate in the inoculum is very recalcitrance to degradation by indigenous microbes and *C. bescii*, also confirmed by the very low methane production in these bottles (data not shown). The microbial changes were also minor when the bottles were fed with untreated substrate. As shown in Fig. 4, the relative abundance of *Dictyoglomus* was increased from 22% in bottles without substrate (inoculum + 0%) to 34% in bottles fed with untreated birch (untreated + 0%). This genus may be responsible for the degradation of part of the carbohydrate fraction of untreated birch. The relative abundance of *Methanothermobacter* and unclassified sequences of the phylum *Methanobacteriaceae* hardly changed when untreated birch was used as substrate compared to inoculum alone.

The bacterial communities were affected substantially when steam-exploded birch was used for biogas production (Figs. 3, 4). The relative abundance of *Firmicutes* increased from 40% in the inoculum + 0% bottles to 62% in the bottles fed with steam-exploded birch without bioaugmentation (pretreated + 0%) (Fig. 5). Whereas the phylum *Dictyoglomi* decreased significantly from 22% in the inoculum + 0% to 3% in the pretreated + 0% bottles. These results indicate that members of *Dictyoglomi* were outcompeted by the *Firmicutes* and the latter likely was responsible for the hydrolysis of steam-exploded birch. Members of the phylum *Firmicutes* were entirely represented by the class *Clostridia,* which previously has been associated with hydrolysis, acidogenesis, and acetogenesis steps [58]. The diversity within *Clostridia* was high, comprising several genera within the dominant orders *Thermoanaerobacterales*, *Clostridiales*, *Natranaerobiales*, and the less abundant orders such as *SHA*-*98* and *OPB54* (Fig. 4). Sequences belonging to the uncultured order *OPB54* accounted for 3.7% of the total reads in the pretreated + 0% bottles, but below 1% in the inoculum + 0% bottles. *OPB54* was previously identified in low abundance in thermophilic laboratory-scale digesters treating stillage [59] and in an enrichment culture amended with lignocellulosic biomass [60]. Culture-independent approaches using DNA-based stable isotopes probing (SIP) revealed that *OPB54* was the most abundant putative SAO bacterium in biogas digesters fed with high levels of acetate [61]. The relative abundance of uncultured-order *SHA*-*98* was lower in the pretreated + 0% bottles compared to the inoculum + 0% bottles, but its function in the methanogenic environment is still unknown. Within the class *Clostridia*, the genus *Ethanoligenens*, unclassified members of the family *ML1228J*-*1,* and *Ruminococcaceae* were detectable in all bottles except those fed with steam-exploded birch.

The relatively higher abundance of members of *Clostridiales* in the bottles fed with steam-exploded birch suggests their role in the hydrolysis of the carbohydrate fraction of steam-exploded birch. Members of *Clostridiales* are ubiquitous in biogas digesters operating with mono- and co-digestion of lignocellulosic materials [51, 52, 62] and have been reported as the main cellulose degrader in biogas digesters [63]. Within the order *Clostridiales*, the abundance of the genus *Clostridium* increased from 3% in the inoculum + 0% to 10% in the pretreated + 0% bottles, whereas the genus *Caldicoprobacter* remained stable (around 3.6%) (Fig. 4). *Caldicoprobacter* contains several xylanolytic bacteria capable of fermenting various sugars into acetate, lactate, ethanol, H_2,_ and CO_2_ [64]. Some members of the *Clostridium* are known syntrophic acetate oxidizing bacteria (SAOB), like the mesophile *C. ultunense* [65]. Other members of the *Clostridium* contain the formyltetrahydrofolate synthetase (FTHFS)-encoding gene for formyltetrahydrofolate synthetase, which is a key enzyme involved in reductive acetogenesis and also the reverse reaction (i.e., SAO), suggesting their SAO capability [66]. In the absence of detectable amount of acetoclastic methanogens in the bottles fed with steam-exploded birch, members of *Clostridium* and other SAOB coupled with hydrogenotrophic methanogens likely contributed to acetate conversion into methane.

The relative abundance of the three genera of the order *Thermoanaerobacterales*, namely, *Coprothermobacter*, *Thermacetogenium,* and *Thermovenabulum,* was increased in the bottles treating steam-exploded birch (Fig. 4). *Coprothermobacter* was the most dominant (41%) genus among all the sequence reads in the pretreated + 0% bottles. Its relative abundance increased by almost 1.6 folds in pretreated + 0% bottles compared to inoculum + 0% bottles. In a previous study of high-rate (HRT of 2–4 days) and high-temperature (60 and 65 °C) anaerobic digesters, *Coprothermobacter* spp. was identified as a main acetate degrader in syntrophic association with hydrogenotrophic *Methanothermobacter* using stable carbon isotopic analysis combined with pyrosequencing methods [12]. *Thermacetogenium* was below 1% in the inoculum + 0% and 1.38% in the pretreated + 0% bottles. *Thermacetogenium phaeum* is a known SAOB [67], which was originally isolated from a thermophilic anaerobic reactor treating kraft-pulp wastewater [68] and found later in many thermophilic biogas digesters [12, 44, 69, 70]. Overall, the higher abundance of bacteria capable of SAO (*Clostridium*, *Coprothermobacter*, *Thermacetogenium,* and uncultured order *OPB54*) as well as the hydrogenotrophic *Methanothermobacter* (see the discussion below) in these thermophilic bottles fed with steam-exploded birch suggests the importance of SAO-HM for efficient degradation of steam-exploded birch into ultimately methane.

As discussed above, the archaeal sequences detected in all thermophilic bottles belonged to only the hydrogenotrophic *Methanobacteriaceae* (Fig. 5), but there were differences in the relative abundance of the sequences at genus level (Fig. 4). *Methanothermobacter* was the dominant genus in the bottles fed with steam-exploded birch with and without bioaugmentation, whereas most of the reads (30–37%) in the rest of bottles were not affiliated to a known genus of the *Methanobacteriaceae.* These changes in archaeal community at genus level are less clear, but the availability of the hydrogenotrophic substrates (H_2_ and CO_2_) due to the improved degradation of pretreated materials (Fig. 1) could give a competitive advantage for the *Methanothermobacter* to dominate in bottles fed with steam-exploded birch. Members of *Methanothermobacter* have been reported as a dominant methanogens in thermophilic biogas digesters (55–65 °C) [12, 44, 55].

Although *C. bescii* was not detectable in the bioaugmented bottles at the final day of the experiment, the microbial community structures were clearly affected by the bioaugmentation (Fig. 4). The relative abundance of the microbial communities of the bottles treating pretreated materials with (pretreated + 10 and + 15%) and without bioaugmentation (pretreated + 0%) was relatively similar, but differs from those bottles bioaugmented with minimum dosages of *C. bescii* (pretreated + 2 and + 5%). For instance, the relative abundance of *Methanothermobacter* was higher in the pretreated + 2% and pretreated + 5% bottles, which reached up to 33–38%, compared to the other bottles at lower abundance (13–27%). The abundance of the known hydrolytic bacterium *Caldicoprobacter* was also higher by twofold in these bioaugmented bottles (pretreated + 2 and + 5%) compared to the others (pretreated + 10 and + 15%). The increase in the abundance of the hydrogenotrophic *Methanothermobacter* and the hydrolytic bacterium *Caldicoprobacter* was well correlated with higher methane production (Table 3 and Fig. 1), suggesting that minimum amount of *C. bescii* was needed to influence the microbiota for efficient degradation of steam-exploded materials into ultimately methane. The reason why the relative abundance of the microbial communities of the bottles fed with pretreated birch with applied higher dosage of bioaugmentation (10 and 15%) and without bioaugmentation was similar and differs from lower bioaugmentation dosage (2 and 5%) is unclear.

There were also changes in bacterial community capable of SAO in bottles fed with pretreated substrate with and without bioaugmentation (Fig. 4). For instance, the abundance of *Coprothermobacter* was significantly reduced from 36 to 41% in the pretreated + 0%, pretreated + 10% and pretreated + 15% bottles to 10–22% in the pretreated + 2% and pretreated + 5% bottles. *Clostridium* was higher in the pretreated + 5% and pretreated + 10% bottles (around 20%) and lower (10–13%) in the rest of the bottles. The decrease and increase of some members of SAOB community without reduction in their combined abundance in these bottles may suggest the functional resilience of the microbial community to temporal changes in the production rate and levels of VFAs. Although we did not measure the concentration of VFAs in each reactor, their concentration and production rate could differ among some of the bottles fed with pretreated material, irrespective of bioaugmentation, as their methane production rate and yield varied (Fig. 1 and Table 3).

Overall, the combined SE pretreatment and bioaugmentation with minimum amount of *C. bescii* contributed to significant increase in methane production (by 140%) and changes in the microbial communities. Interestingly, the bioaugmenting culture (*C. bescii*) was not detectable at the end of the experiment. According to the observed higher methane production rate in our bioaugmented bottles after 9 days (Fig. 1), we speculated that *C. bescii* remained active over 9 days before it was finally outcompeted by the indigenous microbes for nutrients or due to environmental stress (e.g., different pH, VFAs) [14]. Similar results have been reported in a previous bioaugmentation study, where the bioaugmenting culture (*P. xylanivorans Mz5T*) was not detectable at the end of the experiment, while the methane production was improved by 18% and microbial community was affected during bioaugmentation [43].

The enhanced methane production rate and yield by adding only small amounts of the *C. bescii* culture is particularly suitable for upscaling the bioaugmentation process as the cost of cultivating *C. bescii* culture then is limited. Of course, the implication of the present findings for lab-scale semi-continuously fed digesters such as CSTRs needs to be investigated further before scaling up to industrial biogas plants. The challenges of microbial washout particularly in CSTRs and out-competition by indigenous microorganisms during bioaugmentation process should be addressed in future studies to favour survival and prolonged activity of the exogenous (bioaugmenting) microbes. A recent study by Kovacs et al. used a hydrolytic bacterium from the same genus as our study (i.e., *Caldicellulosiruptor saccharolyticus*) to enhance biogas production in CSTRs’ running with a mixture of pig slurry (25% w/v) and chopped sweet sorghum (75% w/v) [21]. Under similar organic loading rate (OLR, 4 g total organic solids/L/day), the addition of *C. saccharolyticus* led to enhanced biogas production, similar to the results obtained in our batch experiments. However, the concentration of the bioaugmenting bacterium was gradually reduced and finally disappeared after 2–3 weeks. Bioaugmentation at the higher OLR of 8-g total organic solids/L/day gave the same beneficial effect of enhanced biogas production as at the lower OLR without dilution of *C. saccharolyticus* over the monitored 24 days. The study demonstrates that changing operating conditions like OLR can be employed as a simple and economical strategy to favour the survival and prolonged activity of the bioaugmenting microbes without the need to carry out the bioaugmentation again and again.

## Conclusions

Methane production was increased up to 140% following pretreatment of birch with steam explosion and bioaugmentation with the cellulolytic *C. bescii*. The enhanced methane production also well correlated with the increase in abundance of key bacterial and archaeal communities, particularly the hydrolytic bacterium *Caldicoprobacter*, several members of SAOB, and the hydrogenotrophic *Methanothermobacter*. Although *C. bescii* was not detectable at the end of the experiment, its activity lasts long enough to improve the degradation of steam-explode birch and ultimately enhanced methane production. This study demonstrates the advantages of combined SE pretreatment and bioaugmentation strategies for successfully reducing biomass recalcitrance and improving hydrolysis, and thus, enhancing methane production from birch, a strategy that may also work for other types of lignocellulosic biomass.

## Abbreviations

AD: anaerobic digestion
CSTR: continuously stirred tank reactor
DGS: distiller grains with solubles
DM: dry matter
FTHFS: formyltetrahydrofolate synthetase
GC: gas chromatography
HM: hydrogenotrophic methanogenesis
HPAEC-PAD: high-performance anion-exchange chromatography with pulsed amperometric detection
LCC: lignin–carbohydrate complex linkages
LMW: lower molecular weight
OLR: organic loading rate
OTUs: operational taxonomic sequences
PCOD: particulate chemical oxygen demand
PCR: polymerase chain reaction
QIIME: Quantitative Insight into Microbial Ecology
RMSE: root mean square error
SAO: syntrophic acetate oxidation
SAOB: syntrophic acetate oxidizing bacteria
SCOD: solubilized chemical oxygen demand
SE: steam explosion
SIP: stable isotope probing
SRA: Sequence Read Archive
TCD: thermal conductivity detector
VS: volatile solids
